# Initial and residual benefits of soil amendments in reducing phosphorus release from soils with simulated snowmelt flooding

**DOI:** 10.1002/jeq2.70151

**Published:** 2026-02-15

**Authors:** Darshani Kumaragamage, Ahmed Lasisi, Madelynn Perry, Douglas Goltz, Nora Casson, Srimathie Indraratne, Inoka Amarakoon

**Affiliations:** ^1^ Department of Environmental Studies and Sciences The University of Winnipeg Winnipeg Manitoba Canada; ^2^ British Columbia Ministry of Agriculture and Food Kelowna British Columbia Canada; ^3^ Department of Chemistry The University of Winnipeg Winnipeg Manitoba Canada; ^4^ Department of Geography The University of Winnipeg Winnipeg Manitoba Canada; ^5^ Department of Soil Science University of Manitoba Winnipeg Manitoba Canada

## Abstract

In the Canadian prairies, spring snowmelt occurs rapidly and causes flooding in low‐lying areas, inducing anaerobic soil conditions and exacerbating phosphorus (P) release to meltwater. Soil amendments can mitigate P loss from flooded soils soon after amendment application; however, their residual benefits are less understood. We examined the initial and residual benefits of alum (Al_2_(SO_4_)_3_·18H_2_O), gypsum (CaSO_4_·2H_2_O), and Epsom salt (MgSO_4_·7H_2_O) in a simulated snowmelt flooding experiment. Intact soil columns were taken from amended and unamended field plots in the same year and 1 year after the amendment application. The soil columns were flooded and incubated at a cold temperature. Porewater and floodwater samples were analyzed for dissolved reactive P (DRP), calcium (Ca), magnesium (Mg), iron (Fe), and manganese (Mn) concentrations, and pH. During the year of application, alum, gypsum, and Epsom salt decreased the mean porewater DRP by 68%, 29%, and 19%, and floodwater DRP by 69%, 51%, and 31%, respectively, relative to unamended treatment, with only alum showing significant differences. One year after applications, alum significantly decreased porewater DRP by 35%, but not floodwater DRP, whereas gypsum or Epsom salt did not decrease porewater or floodwater DRP. Correlation and principal component analysis revealed that porewater and floodwater DRP are positively related to pH and Fe, but only in alum‐amended treatment, suggesting the influence of pH and Fe in stabilizing P. While alum was effective in mitigating P loss from flooded soils, its effectiveness decreased over time, with negligible residual benefits a year later.

## Core Ideas

- Alum amendment decreased P release from soils under simulated snowmelt flooding in the year of application.
- Gypsum and Epsom salt were ineffective in reducing P loss under simulated snowmelt flooding in this soil.
- The effectiveness of alum in mitigating P loss from soil decreased with time after amendment application.
- Residual benefits of amendments in reducing P loss from soils under simulated snowmelt flooding were negligible.

## Plain Language Summary

Phosphorus (P) is an essential plant nutrient. Farmers supply P to crops as fertilizers and manure. However, P can be lost from croplands to water bodies such as lakes. High P concentrations in water bodies can result in algal blooms with adverse environmental effects. In the Canadian prairies, most P gets into water bodies with snowmelt runoff. Soil amendments can retain P in soil and reduce P loss from land; however, their long‐term effectiveness is unclear. We evaluated potential P loss to snowmelt from intact soil columns collected from field plots, which were either unamended or amended with alum, gypsum, or Epsom salt. Two simulated snowmelt flooding studies were conducted in the same year of the amendment application and the following year. Our results showed that alum was effective in reducing P loss from soils to snowmelt. However, its effectiveness decreased over time, with negligible benefits a year later.

**Abbreviations**
DRP: dissolved reactive P
PCA: principal component analysis

## 1 INTRODUCTION

Phosphorus (P) losses from agricultural soils and subsequent P loading into freshwater bodies trigger algal blooms and impair water quality (Schindler, 1977; Schindler et al., 2012). With long‐term fertilizer and manure applications to agricultural soils, P may accumulate over the years and become a major source of P entering water resources, degrading water quality (Kleinman et al., 2011; Motavalli & Miles, 2002). Transport of dissolved and particulate forms of P from agricultural lands to waterways can occur via different pathways, including erosion, runoff, and leaching (Hansen et al., 2002; Kumaragamage & Akinremi, 2018).

In the Canadian prairies, snowfall generally constitutes about one‐third of the annual precipitation (Shook & Pomeroy, 2012), which accumulates over the winter on agricultural lands and can rapidly melt in the spring, causing major snowmelt flooding events (Schindler et al., 2012). Spring snowmelt flooding is the main driver of P losses from agricultural lands in the prairies (Liu et al., 2019; Schindler et al., 2012). Prolonged flooding can cause anaerobic soil conditions, exacerbating P release from the soil into the overlying floodwater (Amarawansha et al., 2015; Kumaragamage et al., 2019; Schärer et al., 2009). Within as little as a few hours after flooding, microorganisms in flooded soils will consume substantial amounts of dissolved oxygen (Ponnamperuma, 1972), making the soil environment anaerobic. Microbial communities capable of using electron acceptors other than oxygen will become more prevalent as the soil redox potential decreases (Smith et al., 2021). Thus, a shift in microbial communities occurs, and the microbially mediated reductive dissolution of nitrate (NO_3_^−^), manganese (Mn^3+^ and Mn^4+^), and iron (Fe^3+^) compounds begins (Barcala et al., 2023). These reductive dissolution processes release significant amounts of P from flooded soils to porewater and overlying floodwater (Amarawansha et al., 2015; Concepcion et al., 2021; Shenker et al., 2005).

While phosphate compounds associated with Mn and Fe are redox‐sensitive, those associated with cations such as calcium (Ca^2+^) and magnesium (Mg^2+^) are not. However, the solubilities of the P compounds associated with Ca^2+^ and Mg^2+^ (Ca‐P and Mg‐P) are affected by changes in pH. The pH‐dependent solubility of P compounds is an important consideration in flooded soils, as prolonged flooding can also impact soil pH (Ponnamperuma, 1972). In alkaline soils low in Fe and Mn, flooding typically causes the pH to decrease due to the buildup of carbon dioxide (CO_2_). Accumulated CO_2_ reacts with water to form carbonic acid (H_2_CO_3_), and lowers the pH toward neutral (Ponnamperuma, 1972). The pH decrease in alkaline soils with flooding may enhance the solubility of both Ca‐P and Mg‐P species, subsequently releasing P into pore water and floodwater (Jayarathne et al., 2016; Scalenghe et al., 2012).

Soil amendments can stabilize P in soils through various mechanisms in flooded conditions and reduce P losses to overlying floodwater (Dharmakeerthi, Kumaragamage, Goltz, et al., 2019; Dharmakeerthi, Kumaragamage, Indraratne, et al., 2019; Kumaragamage et al., 2022; Van et al., 2022; Vitharana et al., 2021). Dharmakeerthi, Kumaragamage, Goltz, et al. (2019) found that in gypsum‐amended flooded soils, the decrease in redox potential below +200 mV was delayed compared to unamended soil and thereby inhibited the microbially mediated reductive dissolution of P compounds associated with Mn and Fe (Mn‐P and Fe‐P). Additionally, the application of amendments to soil may affect pH (Ann et al., 2000). For pH‐sensitive P species, such as hydroxyapatite [Ca_10_(PO_4_)_6_(OH)_2_], an increase or stabilization in pH could inhibit dissolution reactions (Ponnamperuma, 1972). Soil amendments can also directly reduce P losses from the soil through precipitation reactions with the added ions (Attanayake, Dharmakeerthi, et al., 2022; Attanayake, Kumaragamage, et al., 2022; Kumaragamage et al., 2024, 2022), as well as through increased ionic strength, which can increase P sorption (Huang et al., 2013; Ollikainen et al., 2024).

Previously, researchers have found that the use of soil amendments reduced P release from soil to porewater and overlying floodwater to varying degrees from intact soil columns in controlled incubation experiments (Dharmakeerthi, Kumaragamage, Goltz, et al., 2019; Dharmakeerthi, Kumaragamage, Indraratne, et al., 2019; Kumaragamage et al., 2022; Vitharana et al., 2021) and in cold spring weather conditions (Lasisi, Weerasekara, et al., 2023). A field study with fall‐applied gypsum, alum, and Epsom salt plots showed reductions in dissolved reactive P (DRP) concentrations and loads in snowmelt in the spring (6 months after amendment application), with only Epsom salt showing a significant decrease compared to the unamended treatment (Lasisi, Kumaragamage, et al., 2023). However, using the same plots without further application of amendments, Perry et al. (2024) observed that none of the amendments reduced the DRP loads in the following snowmelt season, 18 months after amendment application. Both Lasisi, Kumaragamage, et al. (2023) and Perry et al. (2024) noted that snowmelt accumulation and snowmelt volume were highly variable among plots, potentially resulting in varying degrees of flooding and redox status, which influence both the P release and effectiveness of amendments. However, none of the previous studies evaluated the direct and residual benefits of fall‐applied soil amendments in mitigating P loss from soils under continued flooded conditions at cold temperatures. In the above context, this study was conducted to better understand the initial and residual benefits of amendments in reducing dissolved P loss to snowmelt floodwater. Experiments were carried out under controlled and simulated flooded conditions, using intact soil columns from amended and unamended field plots 2 weeks and 1 year after amendment application. We hypothesized that (a) amendments would provide initial and residual benefits in reducing P loss to snowmelt and (b) the initial benefits of amendments in reducing P loss would be greater than the residual benefits.

## 2 MATERIALS AND METHODS

### 2.1 Site location and description

Soil columns were extracted from a field site in the Red River Basin, southeast of Winnipeg (49° 32′ N, 96° 51′W) near Randolph, Manitoba. The soil at the field site belongs to the Osborne series, classified as Rego Humic Gleysol (Government of Manitoba, 2010) with US soil taxonomy equivalent of Mollic Gleysol (Soil Survey Staff, 2014). This agricultural field has a history of manure application and received liquid swine manure at a rate of about 140,000 L ha^−1^ (supplying approximately 75 kg ha^−1^ total P) 3 days before the study setup (Lasisi, Kumaragamage, et al., 2023). The soil was clay in texture, calcareous, with a pH of 7.7, electrical conductivity (saturated extract) of 4.3 dS m^−1^, organic matter content of 75 g kg^−1^, Olsen P of 80 mg kg^−1^, and a CEC of 69.7 cmol_c_ kg^−1^. Further details of soil characterization are provided in Lasisi, Kumaragamage, et al. (2023).

**Core Ideas**

- Alum amendment decreased P release from soils under simulated snowmelt flooding in the year of application.
- Gypsum and Epsom salt were ineffective in reducing P loss under simulated snowmelt flooding in this soil.
- The effectiveness of alum in mitigating P loss from soil decreased with time after amendment application.
- Residual benefits of amendments in reducing P loss from soils under simulated snowmelt flooding were negligible.

### 2.2 Controlled flooding study

Controlled flooding studies were conducted using intact soil columns from field plots established in the fall of 2020. Details of the field have been elaborated in Lasisi, Kumaragamage, et al. (2023). In brief, the field had 16 plots arranged in a randomized complete block with four treatments and four replications. The treatments were (i) alum (Al_2_(SO_4_)_3_·18H_2_O) amendment, (ii) gypsum (CaSO_4_·2H_2_O) amendment, (iii) Epsom salt (MgSO_4_·7H_2_O) amendment, and (iv) unamended or control treatment. These amendments were selected based on our previous laboratory studies with calcareous soils that showed their effectiveness in reducing dissolved P loss to floodwater (Kumaragamage et al., 2022; Lasisi, Weerasekara, et al., 2023; Vitharana et al., 2021). Each plot was 3 m x 1 m in dimension, with 0.5 m alley between plots. Amendments were applied at a rate of 2.5 Mg ha^−1^ and incorporated. The amendment rate was chosen because gypsum and Epsom salt had been previously reported to be effective at a rate of ≤2.5 Mg ha^−1^ (Norton, 2008; Vitharana et al., 2021).

Intact soil columns were taken 2 weeks after the amendment application (October 2020) and 1 year after the amendment application (October 2021). Cylindrical polyvinyl chloride (PVC) plastic tubes (length = 30 cm, internal diameter = 10 cm) were used to collect intact soil columns (height = 15 cm) from each plot. More details on collecting intact soil columns and experimental setup were reported in our previous studies (Kumaragamage et al., 2022; Lasisi, Weerasekara, et al., 2023). Intact columns were immediately transported to the laboratory and stored at 4°C until the controlled flooding experiment was initiated.

The controlled flooding study in 2021 was conducted outdoors in the spring, 5 months after collecting the columns. Soil columns were partially buried in an open field in Winnipeg (49° 47′ N 97° 12′ W) after excavating the top 15 cm of soil. The depth of the excavation was such that the soil surface within the columns aligned with the surface of the surrounding soil. Soil columns were arranged in a randomized complete block design. Two days after the installation, soil columns were flooded with cold, ultrapure water (Milli‐Q; 18 MΩ cm) to a water headspace of 10 cm, and a redox potential probe with a platinum sensor was inserted vertically through the soil surface to a depth of 5 cm to measure redox potential. Soil columns were kept covered and protected from any precipitation events and left under spring atmospheric conditions throughout the study period. The controlled flooding experiment in 2021 lasted only 4 weeks (April 7–May 6), since the air temperatures rose above +10°C by early May. To have a longer duration of flooding, the flooding study of 2022 was conducted in the laboratory inside a walk‐in cooler at +4°C for 7 weeks following the same protocol as our previous studies (Kumaragamage et al., 2022; Vitharana et al., 2021). Soil columns, extracted 1 year after the amendment application, were flooded with cold, ultrapure water (Milli‐Q; 18 MΩ cm) to a water headspace of 10 cm, and a redox probe with a platinum sensor was inserted vertically to a depth of 5 cm.

### 2.3 Sampling and laboratory analysis

On the first day of flooding (day 0), and subsequently, at weekly intervals, porewater and floodwater samples were taken from all soil columns as described by Vitharana et al. (2021) and Kumaragamage et al. (2022). On each sampling day, 20 mL of floodwater was collected through a syringe and immediately filtered through a 0.45‐µm pore size polypropylene syringe filter (Thermo Fisher Scientific). Porewater (20 mL) samples were collected using pre‐installed Rhizon‐MOM samplers (0.15 µm pore size; Rhizosphere Products) by applying suction using a 20‐mL syringe. After each sampling day, ultrapure water was added to maintain the 10 cm head above the soil surface. In 2021, samples were taken around midday when the mean air temperature was above 0°C to ensure no frozen standing water. Samples were not collected on the seventh day after flooding because the mean daily air temperature during that week was below 0°C, and floodwater and porewater remained frozen.

On each sampling day, the redox potential of each column was measured by inserting an Ag‐AgCl reference electrode with KCl electrolyte into the soil‐floodwater interface and the pre‐installed platinum redox probe, allowing the voltmeter to stabilize before taking the reading (Patrick et al., 1996). The recorded Eh values were corrected to the standard hydrogen electrode potential by adding +200 mV relative to the potential of the reference electrode.

Concentrations of DRP were determined within 6 h of collection in both floodwater and porewater samples using the molybdate blue method (Murphy & Riley, 1962) and measuring the absorbance at 882 nm wavelength using a UV‐Vis spectrophotometer (Ultraspec 500 pro). The pH of each sample was measured using a Fisher Accumet AB15 pH meter. Porewater and floodwater samples were then acidified with nitric acid (100 µL, 16 M) and stored until analysis. Porewater and floodwater samples in the 2021 study were analyzed for Ca, Mg, Fe, Al, Mn, and S concentrations using inductively coupled plasma optical emission spectrometry (Thermo iCAP 6500 Duo). In the 2022 study, porewater and floodwater samples were analyzed for Ca, Mg, Fe, and Mn only using flame atomic absorption spectroscopy (AAnalyst 400, PerkinElmer).

### 2.4 Statistical analysis

All statistical analyses were conducted separately for each study using SAS software (SAS Institute Inc, 2014). PROC GLIMMIX for repeated measure analysis was used to test the significant effect of treatment, sampling day, and their interactions on daily DRP concentrations and pH of floodwater and porewater, as well as redox potential, separately for each year. In each model, treatment and sampling day were fixed effects, while block (replicate) was a random effect with sampling day as a repeated factor. Before PROC GLIMMIX, residuals of all data were checked for conformity to the assumptions of normality. Non‐normally distributed data were log‐normally distributed, and log‐normal distribution was specified in their models. Multiple means comparison was performed with the Tukey‐Kramer test. Correlation analysis and principal component analysis (PCA) were conducted using data for both years for each amendment treatment separately to identify variables (porewater and floodwater pH, Ca, Mg, Mn, and Fe concentrations) that are most closely associated with the changes in the DRP concentrations in each amendment treatment using R software (RStudio Team, 2014). We combined data from both years for PCA analysis since the effects of amendments in reducing DRP showed similar trends in both years. The principal components (PCs) provide information on the most meaningful parameters that describe the whole dataset while affording data reduction with a minimum loss of original information and facilitating the interpretation of results. The Kaiser criterion was used to determine the number of retained PCs. The PCA biplot was analyzed, and two PCs were chosen based on eigenvalues and scree plot outputs. For all statistical analyses, significance was determined at *p* = 0.05.

## 3 RESULTS

### 3.1 Environmental conditions

During the 2021 flooding study, daily air temperature ranged from −3.1°C to 12.5°C. On the days when floodwater was sampled, nighttime air temperatures ranged from −0.5°C to 3.2°C, and daytime air temperatures ranged from 6.2°C to 8.6°C. At the specific times when samples were collected, the mean temperatures ranged from 8.2°C to 11.3°C (Figure S1). In contrast, the flooding study of 2022 conducted under a controlled environment had a constant day and night temperature of 4 ± 1°C throughout the flooding period.

### 3.2 Porewater and floodwater DRP concentrations

**FIGURE 1:**
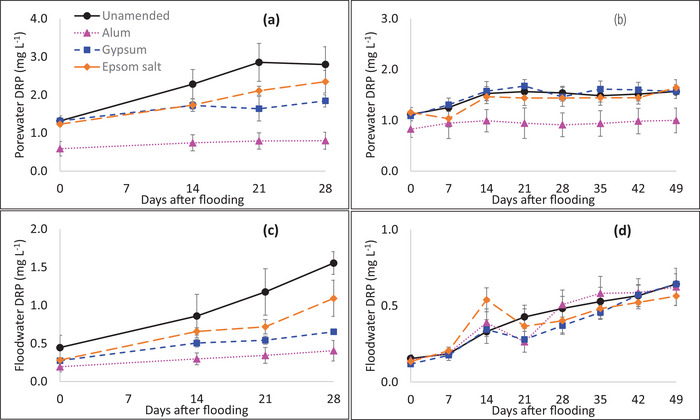
Dissolved reactive P (DRP) concentration changes with time of flooding in porewater from the flooding study of (a) 2021 and (b) 2022 and in floodwater from the flooding study of (c) 2021 and (d) 2022 in unamended, alum‐amended, gypsum amended, and Epsom salt amended treatments. Vertical error bars represent the standard error of the mean.

The results of the 2021 controlled flooding study showed significant treatment (*p* = 0.0004) and flooding time (*p* < 0.0001) effects on porewater DRP concentrations; however, there was no significant interaction of treatment by flooding time (Table S1). The porewater DRP concentrations increased with flooding time in all treatments (Figure 1a). On the day of flooding, the mean porewater concentration across treatments was 1.12 mg L^−1^ and increased to a mean of 1.95 mg L^−1^ by day 28. Averaged across flooding days, the unamended control treatment had the highest porewater DRP concentrations (2.31 mg L^−1^), which was not significantly different from porewater DRP concentrations in gypsum‐amended (1.63 mg L^−1^) and Epsom salt‐amended (1.86 mg L^−1^) treatments. In contrast, the porewater DRP concentrations in the alum‐amended treatment were significantly and on average 68% less (0.73 mg L^−1^) than the unamended control.

The porewater DRP concentrations in the 2022 flooding study were less than in the 2021 study and ranged from 0.82 to 1.65 mg L^−1^. Significant treatment (*p* = 0.0412) and flooding time (*p* < 0.0001) effects were observed for porewater DRP concentrations, but as in the 2021 study, treatment by flooding time interaction was not significant (Table S1). The porewater DRP concentrations increased with time in all treatments (Figure 1b). The mean DRP concentration across treatments increased from 1.04 mg L^−1^ on the day of flooding to 1.44 mg L^−1^ by 7 weeks of flooding. The alum amendment significantly decreased mean porewater DRP concentrations to 0.94 mg L^−1^ (compared to 1.45 mg L^−1^ in the unamended control), resulting in a DRP reduction by 25%–41% depending on the flooding time with an average of 35% across days (Figure 1b). The mean porewater DRP concentrations across days of flooding were not statistically different in unamended (1.45 mg L^−1^), gypsum amended (1.49 mg L^−1^) and Epsom salt‐amended (1.38 mg L^−1^) treatments.

The floodwater DRP concentrations were significantly lower (paired *t*‐test; *p* < 0.0001) than porewater DRP concentrations for all treatments in both years. As with porewater, the floodwater DRP concentrations increased with flooding time (Figure 1c,d). In the 2021 flooding study, treatment (*p* = 0.0003) and flooding time effects (*p* < 0.0001) were significant; however, there was no significant treatment by flooding time interaction (Table S1). The mean floodwater DRP concentrations across treatments were lowest on the day of flooding (0.3 mg L^−1^) and greatest on day 28 (0.93 mg L^−1^) resulting in a threefold increase over 28 days of flooding (Figure 1c). Overall and across time, alum, gypsum, and Epsom salt amendments reduced floodwater DRP concentrations relative to the control treatment by 69%, 51%, and 32%, respectively, but the reduction was significant only in the alum‐amended treatment. In the 2022 flooding study, the floodwater DRP concentrations were less than those in the 2021 study. Contrary to the results of the 2021 study, the treatment effect, flooding time, and the treatment by flooding time interaction were not significant (Figure 1d; Table S1).

### 3.3 Soil redox potential, floodwater pH, and porewater pH

Soil redox potential values were highly variable (data not shown); thus, statistical comparisons were not made. In general, all treatments in both the 2021 and 2022 flooding studies had their highest Eh on the day of flooding, with a mean ± standard deviation of 383 ± 179 mV in the 2021 flooding study and 377 ± 91 mV in the 2022 flooding study (data not shown). The redox potential values decreased in all treatments over the flooding period. By the end of the flooding period, the mean Eh across treatments dropped to 126 ± 105 mV at 28 days of flooding in 2021 and to 258 ± 85 mV at 49 days of flooding in 2022.

**FIGURE 2:**
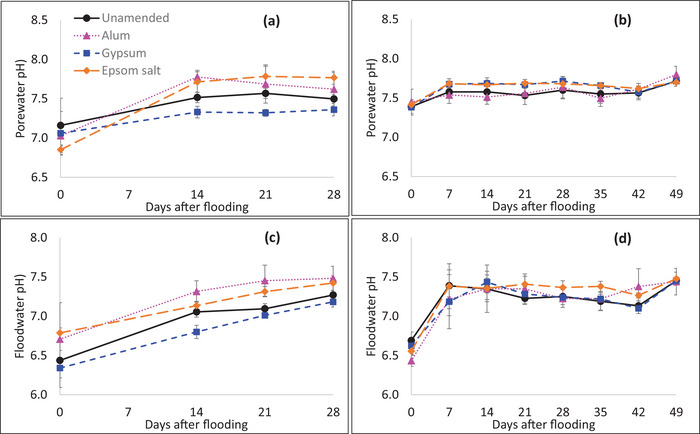
pH changes with time of flooding in porewater from the flooding study of (a) 2021 and (b) 2022, and in floodwater from the flooding study of (c) 2021 and (d) 2022, in unamended, alum‐amended, gypsum amended, and Epsom salt‐amended treatments. Vertical error bars represent the standard error of the mean.

A significant flooding time effect was observed for porewater and floodwater pH; however, the treatment effect and the treatment by flooding time interaction were not significant for floodwater and porewater pH in both years (Table S1). Floodwater and porewater pH increased with the time of flooding in both years (Figure 2). In 2021, the floodwater pH (average across treatments) increased from 6.6 on the day of flooding to 7.3 at 28 days of flooding. In 2022, floodwater pH increased from 6.6 on the day of flooding to 7.5 at 49 days of flooding. The porewater pH was lowest on the day of flooding, with an average (across treatments) of 7.0 and 7.4 in 2021 and 2022, respectively. The porewater pH increased to 7.6 at 28 days of flooding in 2021 and to 7.7 at 49 days of flooding in 2022. A significant increase in floodwater pH was observed at the early stages of flooding, with an average of 7.6 (across treatments) at 14 days of flooding in 2021 and at 7 days of flooding in 2022.

### 3.4 Relationships between porewater and floodwater DRP, cation concentrations, and pH

The concentrations of Ca, Mg, Fe, and Mn in porewater and floodwater varied among the treatments depending on the sampling day (Figures S2–S5). In the 2021 study, porewater samples collected at 21 days of flooding were not analyzed for dissolved Ca, Mg, Fe, and Mn, because the sample volume was small. The porewater Ca and Mg concentrations, in general, decreased with time of flooding (Figures S4 and S5), whereas floodwater Ca and Mg concentrations increased (Figures S2 and S3). The porewater Mn and Fe concentrations did not show a consistent trend with time of flooding in the 2021 study (Figure S4), but in the 2022 study, porewater Mn and Fe decreased initially and then increased at the latter stages of the flooding period (Figure S5). Floodwater Mn concentrations slightly decreased with time of flooding in both 2021 and 2022 studies. Floodwater Fe concentrations did not show a consistent trend in the 2021 study, but in the 2022 study, the concentrations decreased initially before increasing with time toward the end of the flooding period (Figures S2 and S3).

The correlation matrix of porewater and floodwater chemical parameters, when data from all treatments were pooled together and analyzed, reveals that significant, positive correlations exist between porewater and floodwater concentrations of dissolved Ca, Mg, Fe, and Mn (Table S2). However, when data were separated by treatment, significant, positive correlations between porewater and floodwater dissolved Ca, Mg, Fe, and Mn concentrations and pH were shown only in unamended and alum‐amended soils for all parameters (Tables S3 and S4). In gypsum‐ and Epsom salt‐amended treatments, porewater and floodwater pH, DRP, and Fe showed significant and positive correlations, while porewater Ca, Mg, and Mn did not correlate significantly with their respective floodwater concentrations (Tables S5 and S6). The floodwater DRP concentrations in the alum‐amended treatment showed a significant positive correlation with porewater Fe concentrations and porewater pH, which was not observed in other treatments. In the gypsum‐amended treatment, floodwater DRP concentration showed significant and positive correlations with porewater Ca and Mg concentrations (Table S5).

The scree plots describing the amount of variability explained by each PC (Figure S6) and factor loadings (Table 1) were examined to determine the number of components to be retained. According to Kaiser's Rule, three PCs with eigenvalues >1.0 were kept in the unamended and the alum‐amended treatments, capturing 72.8% and 73.6% variation, respectively, whereas four PCs were kept in the gypsum‐ and the Epsom salt‐amended treatments, capturing 82.0% and 80.4% variation, respectively.

**TABLE 1:** Principal component loading matrix of porewater (Pore) and floodwater (Flood) pH, dissolved reactive P (DRP), Ca, Mg, Fe, and Mn concentrations, and variance contribution.

Variable	Unamended			Alum‐amended			Gypsum‐amended				Epsom salt‐amended			
	PC1	PC2	PC3	PC1	PC2	PC3	PC1	PC2	PC3	PC4	PC1	PC2	PC3	PC4
DRP‐Pore	0.004	**0.391**	0.269	0.189	**0.201**	0.180	0.294	0.269	0.114	**0.308**	**0.310**	**0.387**	0.037	0.287
pH‐Pore	−0.234	−0.024	**0.417**	0.166	**0.407**	−0.226	**−0.335**	0.266	0.267	0.104	**0.355**	−0.223	**0.385**	−0.092
Ca‐Pore	**0.443**	−0.042	−0.189	**−0.404**	−0.150	−0.040	**0.342**	−0.219	**−0.371**	0.270	**−0.470**	0.156	−0.015	0.225
Mg‐Pore	**0.432**	0.029	−0.207	**−0.371**	−0.232	−0.017	**0.388**	−0.230	−0.261	0.229	**−0.365**	**0.348**	−0.119	0.235
Mn‐Pore	**0.408**	−0.232	0.187	−**0.362**	0.127	−0.191	−0.053	0.212	**−0.618**	−0.023	−0.263	−0.044	0.119	**0.516**
Fe‐Pore	0.210	−0.110	**0.499**	−0.260	**0.459**	−0.141	−0.230	**0.353**	**−0.378**	0.093	−0.079	−**0.406**	0.252	0.337
DRP‐Flood	−0.157	**0.484**	0.121	0.068	**0.473**	0.039	0.218	**0.454**	0.035	−0.133	**0.410**	0.032	−0.139	0.332
pH‐Flood	−0.110	−0.032	**0.456**	0.237	**0.314**	0.101	−0.163	**0.325**	0.088	**0.476**	0.258	0.063	0.274	**0.334**
Ca‐Flood	**0.373**	0.305	0.147	−0.297	0.201	**0.56**3	**0.346**	0.320	0.031	**−0.346**	0.095	**−0.365**	**−0.504**	0.186
Mg‐Flood	**0.397**	0.281	0.140	−0.307	0.205	**0.529**	**0.357**	0.313	0.005	**−0.359**	0.069	**−0.348**	**−0.512**	0.205
Mn‐Flood	−0.020	−**0.498**	0.003	**−0.362**	−0.007	−0.167	−0.239	−0.215	−0.148	**−0.514**	**−0.278**	**−0.338**	0.074	−0.275
Fe‐Flood	0.131	**−0.351**	**0.356**	−0.251	**0.294**	**−0.470**	**−0.316**	0.170	**−0.391**	−0.047	−0.151	−**0.338**	**0.377**	0.221
**Eigenvalue**	**1.90**	**1.63**	**1.49**	**2.18**	**1.58**	**1.17**	**2.01**	**1.69**	**1.26**	**1.05**	**1.76**	**1.64**	**1.41**	**1,28**
**Variance %**	**30.9**	**22.9**	**19.0**	**40.6**	**21.3**	**11.8**	**34.6**	**24.4**	**13.5**	**9.5**	**26.4**	**22.9**	**17.0**	**14.1**
**Cumulative variance %**	**30.9**	**53.8**	**72.8**	**40.6**	**61.9**	**73.7**	**34.6**	**59.0**	**72.5**	**82.0**	**26.4**	**49.3**	**66.3**	**80.4**

*Note*: Variables with the highest loadings are indicated in bold.

**FIGURE 3:**
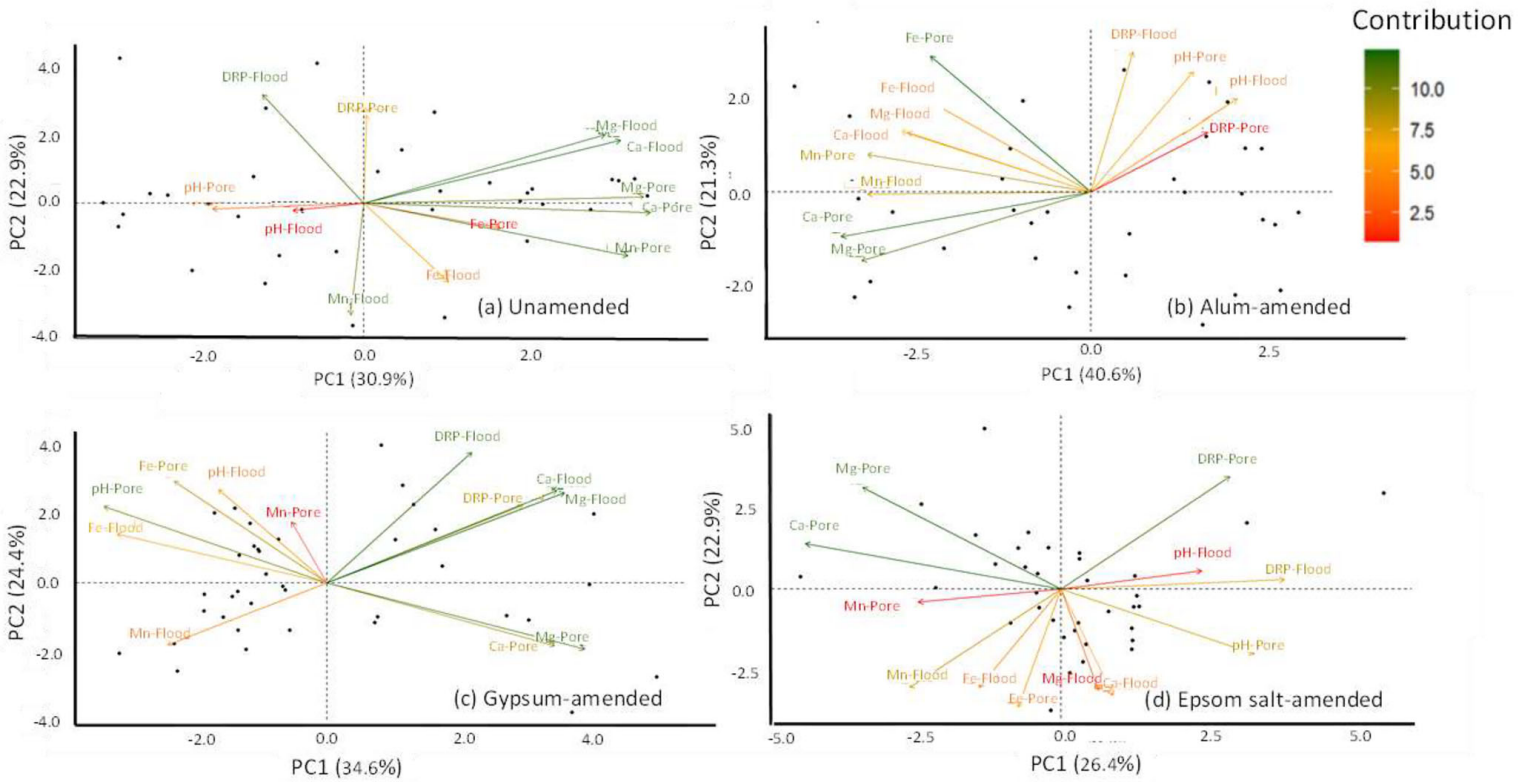
Principal component score and loading plots of porewater (Pore) and floodwater (Flood) dissolved reactive P (DRP), Ca, Mg, Fe, and Mn concentrations, and pH in (a) unamended, (b) alum‐amended, (c) gypsum‐amended, and (d) Epsom salt‐amended treatments. The results are displayed by the biplot, and the parentheses of principal component analysis (PCA) are the percentages of variation.

The PCA biplots of all four treatments are shown in Figure 3. In the unamended treatment, both floodwater Ca and Mg concentrations and porewater Ca and Mg concentrations showed highly correlated pairings, and together with porewater Mn concentrations, they showed strong positive loadings to PC1, accounting for 30.8% variation (Table 1; Figure 3). The PC2, accounting for 22.9% variation, grouped porewater and floodwater DRP concentrations positively, which were inversely associated with floodwater Fe and Mn concentrations. In the alum‐amended treatment, porewater Mg, Ca, and Mn concentrations and floodwater Mn concentrations showed strong negative loadings to PC1, which accounted for 40.1% variation, while porewater and floodwater DRP concentrations and pH, together with porewater Fe concentrations, were positively correlated with PC2, accounting for 21.3% variation. In the gypsum‐amended treatment, PC1 grouped Ca and Mg concentrations in both porewater and floodwater, which were inversely associated with porewater pH and floodwater Fe concentration, and accounted for 34.6% variation. The PC2, which accounted for 24.4% variation, grouped floodwater pH, DRP, and porewater Fe concentrations positively. In Epsom salt treatment, porewater and floodwater DRP concentrations and pH had positive loadings to PC1, with porewater Ca, Mg, and floodwater Mn concentrations with strong negative loadings, accounting for 26.4% variation. The PC2, which accounted for 22.9% variation, grouped porewater DRP and Mg concentrations positively and porewater Fe and floodwater Ca, Mg, Mn, and Fe concentrations negatively (Table 1; Figure 3).

## 4 DISCUSSION

### 4.1 Change in DRP concentrations with time

The significant increase in DRP concentrations in porewater and overlying floodwater from intact columns with the length of flooding, irrespective of the treatment in both the 2021 and 2022 studies, clearly showed that prolonged flooding during the snowmelt period poses a potential threat of increasing P transport to water bodies. This is likely due to the anaerobic conditions created in soils by overlying floodwater, which enhances P release due to reductive dissolution reactions as previously reported (Amarawansha et al., 2015; Vitharana et al., 2021). Using flooded, intact columns from agricultural soils in the same region, Attanayake, Kumaragamage, et al. (2022) showed that reductive dissolution of Fe (III) oxy(hydr)oxides and subsequent release of associated P was the main reason for enhanced DRP concentration in porewater.

Contrary to the typical response of alkaline soils to gradually shift pH toward neutrality due to CO_2_ accumulation when flooded for a prolonged period (Amarawansha et al., 2015; Ponnamperuma, 1972), the porewater pH increased in the current study. Cold temperatures in the current study may have suppressed microbial respiration, thereby reducing CO_2_ accumulations, which could be the reason for not observing a pH decrease, as previously observed with alkaline soils flooded under cold temperatures (Kumaragamage et al., 2020). The increase in pH may have influenced DRP concentrations, since the initial rise in porewater DRP concentration stabilized over time, most likely due to reprecipitation.

The DRP concentrations in both porewater and floodwater were lower in the 2022 study compared to the 2021 study. In the 2021 study, the field plots received manure in the fall of 2020, 2 weeks before soil columns were extracted, whereas no further manure was applied in the following year, which could be one reason for the greater DRP concentrations in the 2021 study. In addition, the differences in DRP concentrations could be due to the temperature differences during the flooding period between the two studies. In 2021, the experiment was conducted outdoors during the spring, and the mean daily temperatures often exceeded 10°C, while nighttime negative temperatures caused freeze‐thaw events. In contrast, the 2022 study was under a controlled environment with a constant day and night temperature of 4 ± 1°C throughout the flooding period. This may have resulted in a much larger P release to floodwater in the 2021 study than the 2022 study, similar to previous findings where P release from flooded soils was greater under warm conditions compared to cold (Kumaragamage et al., 2020; Weerasekara et al., 2021). Despite the low temperatures and freeze‐thaw events during the night when the temperature fell below 0°C, the P dissolution in the 2021 study was not impaired. Enhanced P release from soils to floodwater has been documented under summer (or warm temperature) flooding, as well as under simulated spring (cold temperature) conditions (Kumaragamage et al., 2020; Lasisi, Weerasekara, et al., 2023; Vitharana et al., 2021), as observed in the current study. Compared to floodwater, consistently higher DRP concentrations in porewater are a clear indication that P gets initially released to porewater from soils and subsequently diffuses into floodwater. As such, management practices, such as the application of soil amendments that interact with soil constituents in reducing P release to porewater, are viable options to reduce potential P release to floodwater or runoff in regions where P loss is not erosion‐driven.

### 4.2 Amendment effects on DRP concentration

Alum amendment reduced the porewater and floodwater DRP concentrations significantly in the 2021 study by 68% and 72%, respectively, and in the 2022 study, a significant decrease of 35% was observed for porewater DRP concentration. However, in field studies conducted in 2021 and 2022, where snowmelt DRP concentrations and loads were measured in the same amended field plots from which intact columns were collected for the current study, a significant decrease in snowmelt DRP concentration by alum amendment was not observed (Lasisi, Weerasekara, et al., 2023; Perry et al., 2024). This could be partly because of the non‐flooding conditions in the field compared to continuous flooding conditions maintained in the more controlled column study. The lack of a significant reduction in snowmelt DRP concentration with alum amendment in the field study could have also been due to the high variability in snowmelt volume among replicates (Lasisi, Kumaragamage, et al., 2023; Perry et al., 2024), which was not a factor in the laboratory‐controlled study.

Effectiveness of alum in reducing flooding‐induced P release from soils has been previously reported with a 43%–73% reduction in porewater DRP concentrations and 27%–64% reduction in floodwater DRP concentrations across eight soil columns from agricultural lands, flooded and kept outside during spring conditions, but unburied (Lasisi, Weerasekara, et al., 2023). The effects of gypsum and Epsom salt on the release of P from soil to porewater were not significant in both the 2021 and 2022 studies. Past research has shown significant decreases with the use of these amendments; however, these experiments were conducted shortly after amendment application, where amendments were directly applied to the surface of intact soil columns or mixed with soils before packing in packed column studies (Dharmakeerthi, Kumaragamage, Goltz, et al., 2019; Dharmakeerthi, Kumaragamage, Indraratne, et al., 2019; Kumaragamage et al., 2022; Vitharana et al., 2021). In a laboratory intact soil column study comparing gypsum and alum applied at the rate of 5 Mg ha^−1^, which is double the rate used in the current study for field application, Kumaragamage et al. (2022) observed that alum showed a greater and more consistent effect across different soil types in reducing P release from flooded columns to floodwater.

In the 2021 study conducted within a year of amendment application, alum amendment decreased P release from flooded soil columns; however, a year after the amendment application in the 2022 study, the effectiveness of alum in decreasing P release from soils was greatly reduced. Differences in environmental conditions between 2021 and 2022, particularly temperature, may have influenced DRP concentrations in porewater and floodwater; however, prior studies suggest that amendment efficacy is unlikely to be temperature dependent. For example, previous laboratory experiments using intact soil columns treated with the same three amendments and subjected to flooding at a constant temperature of +4°C shortly after amendment application showed significant reductions in both porewater and floodwater DRP concentrations across most of the tested soils (Kumaragamage et al., 2022; Vitharana et al., 2021). Our results, therefore, imply that the effectiveness of amendments in stabilizing soil P is greatest soon after application, and over time, they lose their ability to stabilize P in soils and reduce P losses.

Several mechanisms contribute to reduced P release from flooded soils following the application of a chemical amendment. In alkaline or calcareous soils, chemical amendments can increase soil solution Ca levels, either by directly supplying Ca (e.g., gypsum) or by displacing exchangeable Ca^2+^ with added cations (e.g., Al^3+^ from alum), promoting Ca‐P precipitation (Attanayake, Dharmakeerthi, et al., 2022; Kordlaghari & Rowell, 2006; Kumaragamage et al., 2022; 2024). Prior studies using P K‐edge X‐ray absorption near‐edge structure spectroscopy showed a greater proportion of apatite‐like P and a correspondingly lower proportion of P‐sorbed to calcite (CaCO_3_) in alum‐ and Epsom salt‐amended soils compared to unamended soils from the same amended plots as the current study (Kumaragamage et al., 2024). Backscattered imaging from scanning electron microscopy‐energy dispersive X‐rays further revealed P‐enriched microsites associated with Al, Fe, Mg, and Ca in these treatments (Kumaragamage et al., 2024). In calcareous soils, alum also enhanced the Al‐ and Fe‐bound P fraction via adsorption to poorly crystallized Al hydr(oxides) and ferric phosphate precipitation (Fan et al., 2019). In the current study, correlation and PCA indicated positive relationships between DRP, pH, and Fe concentrations in alum‐treated soils, suggesting that Fe‐associated P is likely a pH‐sensitive source of released P. Additionally, it would be expected that the ionic strength of a soil solution would be increased with the addition of any of the amendments. Increased ionic strength in amended soils may have enhanced P adsorption, further limiting its release to overlying water (Liu et al., 2011; Murphy & Stevens, 2010).

Gypsum and Epsom salt amendments were ineffective in stabilizing P in this soil and did not significantly decrease floodwater DRP concentrations. Previous research has shown that gypsum and Epsom salt are effective in reducing P release from flooded soils, but the effectiveness is highly dependent on soil properties (Dharmakeerthi, Kumaragamage, Indraratne, et al., 2019; Kumaragamage et al., 2022; Vitharana et al., 2021). Using Visual MINTEQ software, Attanayake, Dharmakeerthi, et al. (2022) predicted that P stabilization with gypsum amendment may occur through β‐tricalcium phosphate and octacalcium phosphate precipitation, which in turn will reduce DRP concentrations in porewater. Results of correlation analysis and PCA in the gypsum‐amended treatment of the current study corroborate these predictions, showing strong positive relationships between Ca and Mg concentrations with DRP concentrations. However, the soil used in the current study was calcareous with excessive Ca concentrations; thus, further increases in soil solution Ca concentration with gypsum amendment did not significantly influence the DRP concentrations in both 2021 and 2022 studies.

## 5 CONCLUSIONS

Flooding under simulated snowmelt conditions enhanced P release from soils, irrespective of the amendment treatment. Gypsum and Epsom salt were ineffective in reducing P release from soils under simulated snowmelt flooding within a year after the amendment application or 1 year after application. In contrast, alum amendment effectively reduced P release from flooded soil columns; however, the effectiveness decreased with time of application. Our results indicate that amendments likely lose their ability to stabilize P in soils and reduce P losses over time, suggesting frequent application of amendments may be required to mitigate P losses from agricultural soils with snowmelt flooding. To fully understand the capabilities of amendments in reducing P losses from soils under snowmelt conditions, more research using various soil types under field snowmelt flooding conditions is needed.

## AUTHOR CONTRIBUTIONS

**Darshani Kumaragamage**: Conceptualization; data curation; formal analysis; funding acquisition; investigation; methodology; project administration; resources; supervision; validation; visualization; writing—original draft; writing—review and editing. **Ahmed Lasisi**: Conceptualization; data curation; formal analysis; investigation; methodology; validation; visualization; writing—review and editing. **Madelynn Perry**: Conceptualization; data curation; formal analysis; investigation; methodology; validation; visualization; writing—review and editing. **Douglas Goltz**: Conceptualization; formal analysis; funding acquisition; investigation; methodology; resources; supervision; validation; writing—review and editing. **Nora Casson**: Conceptualization; formal analysis; funding acquisition; investigation; methodology; resources; supervision; validation; writing—review and editing. **Srimathie Indraratne**: Conceptualization; formal analysis; funding acquisition; investigation; supervision; writing—review and editing. **Inoka Amarakoon**: Conceptualization; formal analysis; funding acquisition; investigation; supervision; writing—review and editing.

## CONFLICT OF INTEREST STATEMENT

The authors declare no conflicts of interest.

## Supporting information



The supplemental material includes four tables providing information on ANOVA results for porewater and floodwater dissolved reactive P (DRP) concentrations and pH (Table S1), correlation coefficient matrix between porewater and floodwater pH and DRP, Ca, Mg, Fe, and Mn concentrations with pooled data from 2021 and 2022 studies for all treatments (Table S2) unamended (Table S3) alum‐amended (Table S4), gypsum‐amended (Table S5) and Epsom salt‐amended treatments (Table S6). It also contains six figures providing information on diurnal and daily mean air temperature during the experimental period (Fig. S1), floodwater concentrations of (a) Ca, (b) Mg, (c) Mn, and (d) Fe with flooding time in soil columns in unamended, alum‐amended, gypsum amended, and Epsom salt amended treatments from the flooding study of 2021 (Fig. S2) and 2022 (Fig. S3), porewater concentrations of (a) Ca, (b) Mg, (c) Mn, and (d) Fe with flooding time in soil columns in unamended, alum‐amended, gypsum amended, and Epsom salt amended treatments from the flooding study of 2021(Fig. S4) and 2022 (Fig. S5) and Scree plots for PCA analysis in unamended, alum‐amended, gypsum amended, and Epsom salt amended treatments (Fig. S6).

## Data Availability

Data will be made available on request.
