# Supplementary material for: Initial and residual benefits of soil amendments in reducing phosphorus release from soils with simulated snowmelt flooding
Source: J Environ Qual. 2026 Feb 15;55(1):e70151. doi: 10.1002/jeq2.70151 (PMC12907610; doi:10.1002/jeq2.70151)
Supplement: Supplementary file 1 — The supplemental material includes four tables providing information on ANOVA results for porewater and floodwater dissolved reactive P (DRP) concentrations and pH (Table S1), correlation coefficient matrix between porewater and floodwater pH and DRP, Ca, Mg, Fe, and Mn concentrations with pooled data from 2021 and 2022 studies for all treatments (Table S2) unamended (Table S3) alum‐amended (Table S4), gypsum‐amended (Table S5) and Epsom salt‐amended treatments (Table S6). It also contains six figures providing information on diurnal and daily mean air temperature during the experimental period (Fig. S1), floodwater concentrations of (a) Ca, (b) Mg, (c) Mn, and (d) Fe with flooding time in soil columns in unamended, alum‐amended, gypsum amended, and Epsom salt amended treatments from the flooding study of 2021 (Fig. S2) and 2022 (Fig. S3), porewater concentrations of (a) Ca, (b) Mg, (c) Mn, and (d) Fe with flooding time in soil columns in unamended, alum‐amended, gypsum amended, and Epsom salt amended treatments from the flooding study of 2021(Fig. S4) and 2022 (Fig. S5) and Scree plots for PCA analysis in unamended, alum‐amended, gypsum amended, and Epsom salt amended treatments (Fig. S6). [file JEQ2-55-0-s001.docx]

**Supplemental material**

**Initial and Residual Benefits of Soil Amendments in Reducing Phosphorus Release from Soils with Simulated Snowmelt Flooding**

Darshani Kumaragamage^1*^, Ahmed Lasisi^1,2^, Madelynn Perry^1^, Douglas Goltz^3^, Nora Casson^4^, Srimathie Indraratne^1^ and Inoka Amarakoon^5,1^

^1^ Department of Environmental Studies and Sciences, The University of Winnipeg, Winnipeg, MB, Canada R3B 2E9

^2^ British Columbia Ministry of Agriculture and Food, 200-1690 Powick Rd, Kelowna, British Columbia, V1X 7G5, Canada

^3^ Department of Chemistry, The University of Winnipeg, Winnipeg, MB Canada R3B 2E9

^4^ Department of Geography, The University of Winnipeg, Winnipeg, MB Canada R3B 2E9

^5^ Department of Soil Science, University of Manitoba, Winnipeg, MB Canada R3T 2N2

*Corresponding author: [d.kumaragamage@uwinnipeg.ca](mailto:d.kumaragamage@uwinnipeg.ca)

This section contains eleven pages with four tables.

**Supplemental Figure S1**: Diurnal and daily mean air temperature during the experimental period. Red asterisk means sampling dates.

Source: Weather Canada https://climate.weather.gc.ca/

**Supplemental Table S1.** Effect of soil amendments on floodwater and porewater dissolved reactive phosphorus (DRP) concentrations and pH

| Variables | Model effect | 2021 | 2022 |  |
| --- | --- | --- | --- | --- |
| Floodwater DRP concentrations | |  |  |  |
|  | Treatment | 0.0003 | 0.1947 |  |
|  | Sampling day | <.0001 | <.0.001 |  |
|  | Sampling day x Treatment | 0.2125 | 0.9902 |  |
| Porewater DRP concentrations | |  |  |  |
|  | Treatment | 0.0004 | 0.0412 |  |
|  | Sampling day | <.0001 | <.0001 |  |
|  | Sampling day x Treatment | 0.1017 | 0.5346 |  |
| Floodwater pH | |  |  |  |
|  | Treatment | 0.0631 | 0.9802 |  |
|  | Sampling day | <.0001 | <0.0001 |  |
|  | Sampling day x Treatment | 0.9602 | 0.7617 |  |
| Porewater pH | |  |  |  |
|  | Treatment | 0.2142 | 0.6274 |  |
|  | Sampling day | <.0001 | <0.0001 |  |
|  | Sampling day x Treatment | 0.1572 | 0.8721 |  |

**Supplemental Figure S2.** Changes in floodwater concentrations of (a) Ca, (b) Mg, (c) Mn, and (d) Fe with flooding time in soil columns from the flooding study of 2021 in unamended, alum amended, gypsum amended, and Epsom salt amended treatments.

**Supplemental Figure S3**. Changes in floodwater concentrations of (a) Ca, (b) Mg, (c) Mn, and (d) Fe with flooding time in soil columns from the flooding study of 2022 in unamended, alum amended, gypsum amended, and Epsom salt amended treatments.

**Supplemental Figure S4**. Changes in porewater concentrations of (a) Ca, (b) Mg, (c) Mn, and (d) Fe with flooding time in soil columns from the flooding study of 2021 in unamended, alum amended, gypsum amended, and Epsom salt amended treatments. Note: Porewater samples were analyzed for cations only on day zero and day 28, since the sample volume was too small on other days.

**Supplemental Figure S5**. Changes in porewater concentrations of (a) Ca, (b) Mg, (c) Mn, and (d) Fe with flooding time in soil columns from the flooding study of 2022 in unamended, alum amended, gypsum amended, and Epsom salt amended treatments.

**Supplemental Table S2**. Correlation coefficient matrix between porewater and floodwater pH and DRP, Ca, Mg, Fe, and Mn concentrations with pooled data from 2021 and 2022 studies for all treatments.

|  | DRP porewater | pH porewater | Ca porewater | Mg Porewater | Mn porewater | Fe porewater | DRP floodwater | pH floodwater | Ca floodwater | Mg floodwater | Mn floodwater | Fe floodwater |
| --- | --- | --- | --- | --- | --- | --- | --- | --- | --- | --- | --- | --- |
| DRP porewater | 1.00 |  |  |  |  |  |  |  |  |  |  |  |
| pH porewater | 0.14 | 1.00 |  |  |  |  |  |  |  |  |  |  |
| Ca porewater | -0.14 | -0.52*** | 1.00 |  |  |  |  |  |  |  |  |  |
| Mg porewater | -0.07 | -0.58*** | 0.90*** | 1.00 |  |  |  |  |  |  |  |  |
| Mn porewater | -0.20* | -0.03 | 0.50*** | 0.39*** | 1.00 |  |  |  |  |  |  |  |
| Fe porewater | -0.10 | 0.25** | 0.11 | -0.03 | 0.60*** | 1.00 |  |  |  |  |  |  |
| DRP floodwater | 0.46*** | 0.18* | -0.25** | -0.21** | -0.17* | 0.15 | 1.00 |  |  |  |  |  |
| pH floodwater | 0.09 | 0.50*** | -0.35*** | -0.32*** | -0.01 | 0.21** | 0.18* | 1.00 |  |  |  |  |
| Ca floodwater | -0.04 | -0.17* | 0.28*** | 0.24** | 0.28*** | 0.20* | 0.22** | -0.07 | 1.00 |  |  |  |
| Mg floodwater | -0.05 | -0.18* | 0.29*** | 0.27*** | 0.29*** | 0.21** | 0.22** | -0.06 | 0.97*** | 1.00 |  |  |
| Mn floodwater | -0.31*** | 0.01 | 0.20* | 0.08 | 0.27*** | 0.19* | -0.32*** | -0.19* | 0.00 | 0.01 | 1.00 |  |
| Fe floodwater | -0.23** | 0.18* | 0.06 | -0.07 | 0.46*** | 0.70*** | -0.13 | 0.15 | -0.08 | -0.04 | 0.29*** | 1.00 |
|  |  |  |  |  |  |  |  |  |  |  |  |  |
| Note: *, ** and *** denotes significant correlation at P < 0.05, 0.01 and 0.001, respectively. Significant positive correlations are shaded in green and significant negative correlations are shaded in orange. | | | | | | | | | | | | |

**Supplemental Table S3**. Correlation coefficient matrix between porewater and floodwater pH and DRP, Ca, Mg, Fe, and Mn concentrations with pooled data from 2021 and 2022 studies for unamended (control) treatment.

|  | DRP porewater | pH porewater | Ca porewater | Mg Porewater | Mn porewater | Fe porewater | DRP floodwater | pH floodwater | Ca floodwater | Mg floodwater | Mn floodwater | Fe floodwater |
| --- | --- | --- | --- | --- | --- | --- | --- | --- | --- | --- | --- | --- |
| DRP porewater | 1.00 |  |  |  |  |  |  |  |  |  |  |  |
| pH porewater | 0.31* | 1.00 |  |  |  |  |  |  |  |  |  |  |
| Ca porewater | -0.03 | -0.48** | 1.00 |  |  |  |  |  |  |  |  |  |
| Mg porewater | -0.06 | -0.50*** | 0.91*** | 1.00 |  |  |  |  |  |  |  |  |
| Mn porewater | -0.05 | 0.00 | 0.62*** | 0.47** | 1.00 |  |  |  |  |  |  |  |
| Fe porewater | 0.19 | 0.16 | 0.09 | 0.09 | 0.63*** | 1.00 |  |  |  |  |  |  |
| drp floodwater | 0.66*** | 0.08 | -0.36* | -0.25 | -0.48** | 0.03 | 1.00 |  |  |  |  |  |
| pH floodwater | 0.15 | 0.50*** | -0.22 | -0.32* | 0.06 | 0.33* | 0.05 | 1.00 |  |  |  |  |
| Ca floodwater | 0.26 | -0.14 | 0.40** | 0.43** | 0.44** | 0.29 | 0.14 | 0.04 | 1.00 |  |  |  |
| Mg floodwater | 0.26 | -0.15 | 0.46** | 0.50*** | 0.49** | 0.30* | 0.11 | 0.01 | 0.98*** | 1.00 |  |  |
| Mn floodwater | -0.358* | 0.02 | 0.11 | 0.05 | 0.36** | 0.10 | -0.55** | 0.04 | -0.35* | -0.29 | 1.00 |  |
| Fe floodwater | -0.18 | 0.12 | 0.05 | 0.03 | 0.46** | 0.65*** | -0.35* | 0.23 | -0.02 | 0.02 | 0.34* | 1.00 |

**Supplemental Table S4**. Correlation coefficient matrix between porewater and floodwater pH and DRP, Ca, Mg, Fe, and Mn concentrations with pooled data from 2021 and 2022 studies for alum-amended treatment.

|  | DRP porewater | pH porewater | Ca porewater | Mg Porewater | Mn porewater | Fe porewater | DRP floodwater | pH floodwater | Ca floodwater | Mg floodwater | Mn floodwater | Fe floodwater |
| --- | --- | --- | --- | --- | --- | --- | --- | --- | --- | --- | --- | --- |
| DRP porewater | 1.00 |  |  |  |  |  |  |  |  |  |  |  |
| pH porewater | 0.27 | 1.00 |  |  |  |  |  |  |  |  |  |  |
| Ca porewater | -0.35* | -0.39* | 1.00 |  |  |  |  |  |  |  |  |  |
| Mg porewater | -0.43** | -0.43** | 0.97*** | 1.00 |  |  |  |  |  |  |  |  |
| Mn porewater | -0.39* | -0.12 | 0.58*** | 0.48** | 1.00 |  |  |  |  |  |  |  |
| Fe porewater | -0.09 | 0.25 | 0.32* | 0.17 | 0.68*** | 1.00 |  |  |  |  |  |  |
| DRP floodwater | 0.34* | 0.40* | -0.25 | -0.34* | 0.05 | 0.42** | 1.00 |  |  |  |  |  |
| pH floodwater | 0.19 | 0.49** | -0.57*** | -0.54*** | -0.29 | 0.03 | 0.27 | 1.00 |  |  |  |  |
| Ca floodwater | 0.07 | -0.20 | 0.45** | 0.37* | 0.50*** | 0.50*** | 0.13 | -0.10 | 1.00 |  |  |  |
| Mg floodwater | -0.09 | -0.19 | 0.47** | 0.40** | 0.52*** | 0.52*** | 0.10 | -0.09 | 0.98*** | 1.00 |  |  |
| Mn floodwater | -0.19 | -0.22 | 0.73*** | 0.65*** | 0.61*** | 0.40** | -0.20 | -0.41** | 0.46** | 0.49** | 1.00 |  |
| Fe floodwater | -0.12 | 0.11 | 0.37* | 0.25 | 0.64** | 0.74** | 0.17 | -0.09 | 0.08 | 0.14 | 0.48** | 1.00 |

**Supplemental Table S5**. Correlation coefficient matrix between porewater and floodwater pH and DRP, Ca, Mg, Fe, and Mn concentrations with pooled data from 2021 and 2022 studies for gypsum-amended treatment.

|  | DRP porewater | pH porewater | Ca porewater | Mg Porewater | Mn porewater | Fe porewater | DRP floodwater | pH floodwater | Ca floodwater | Mg floodwater | Mn floodwater | Fe floodwater |
| --- | --- | --- | --- | --- | --- | --- | --- | --- | --- | --- | --- | --- |
| DRP porewater | 1.00 |  |  |  |  |  |  |  |  |  |  |  |
| pH porewater | -0.14 | 1.00 |  |  |  |  |  |  |  |  |  |  |
| Ca porewater | 0.23 | -0.73*** | 1.00 |  |  |  |  |  |  |  |  |  |
| Mg porewater | 0.27 | -0.78*** | 0.94*** | 1.00 |  |  |  |  |  |  |  |  |
| Mn porewater | -0.04 | 0.24 | 0.05 | -0.08 | 1.00 |  |  |  |  |  |  |  |
| Fe porewater | -0.02 | 0.38* | -0.30 | -0.40** | 0.55*** | 1.00 |  |  |  |  |  |  |
| DRP floodwater | 0.56*** | -0.02 | -0.02 | -0.04 | 0.09 | 0.30 | 1.00 |  |  |  |  |  |
| pH floodwater | 0.06 | 0.64*** | -0.28 | -0.35* | 0.24 | 0.42** | 0.23 | 1.00 |  |  |  |  |
| Ca floodwater | 0.50*** | -0.21 | 0.18 | 0.24 | 0.17 | -0.09 | 0.72*** | -0.07 | 1.00 |  |  |  |
| Mg floodwater | 0.53*** | -0.25 | 0.21 | 0.28 | 0.16 | -0.09 | 0.76*** | -0.08 | 0.97*** | 1.00 |  |  |
| Mn floodwater | -0.60*** | 0.16 | -0.25 | -0.29 | 0.06 | 0.06 | -0.35* | -0.06 | -0.36* | -0.31 | 1.00 |  |
| Fe floodwater | -0.21 | 0.26 | -0.39* | -0.52*** | 0.46** | 0.72*** | -0.03 | 0.16 | -0.33* | -0.32 | 0.16 | 1.00 |

**Supplemental Table S6**. Correlation coefficient matrix between porewater and floodwater pH and DRP, Ca, Mg, Fe, and Mn concentrations with pooled data from 2021 and 2022 studies for Epsom salt-amended treatment.

|  | DRP porewater | pH porewater | Ca porewater | Mg Porewater | Mn porewater | Fe porewater | DRP floodwater | pH floodwater | Ca floodwater | Mg floodwater | Mn floodwater | Fe floodwater |
| --- | --- | --- | --- | --- | --- | --- | --- | --- | --- | --- | --- | --- |
| DRP porewater | 1.00 |  |  |  |  |  |  |  |  |  |  |  |
| pH porewater | 0.09 | 1.00 |  |  |  |  |  |  |  |  |  |  |
| Ca porewater | -0.16 | -0.63*** | 1.00 |  |  |  |  |  |  |  |  |  |
| Mg porewater | 0.09 | -0.74*** | 0.79*** | 1.00 |  |  |  |  |  |  |  |  |
| Mn porewater | -0.07 | -0.17 | 0.43** | 0.33* | 1.00 |  |  |  |  |  |  |  |
| Fe porewater | -0.28 | 0.25 | 0.00 | -0.28 | 0.49** | 1.00 |  |  |  |  |  |  |
| DRP floodwater | 0.69*** | 0.31 | -0.39* | -0.31 | -0.09 | 0.03 | 1.00 |  |  |  |  |  |
| pH floodwater | 0.35* | 0.42** | -0.26 | -0.03 | 0.10 | 0.04 | 0.22 | 1.00 |  |  |  |  |
| Ca floodwater | -0.26 | -0.06 | -0.21 | -0.23 | 0.02 | 0.18 | 0.20 | -0.09 | 1.00 |  |  |  |
| Mg floodwater | -0.27 | -0.11 | -0.15 | -0.16 | 0.03 | 0.17 | 0.26 | -0.07 | 0.98*** | 1.00 |  |  |
| Mn floodwater | -0.53*** | 0.08 | 0.13 | -0.06 | 0.06 | 0.18 | -0.26 | -0.40** | 0.19 | 0.18 | 1.00 |  |
| Fe floodwater | -0.37* | 0.23 | 0.17 | -0.11 | 0.34* | 0.64*** | -0.22 | 0.19 | -0.01 | -0.01 | 0.19 | 1.00 |

**Supplemental Figure S6**. Scree plots for PCA analysis in unamended, alum amended, gypsum amended, and Epsom salt amended treatments.
